# Antimicrobial resistance control efforts in Africa: a survey of the role of Civil Society Organisations

**DOI:** 10.1080/16549716.2020.1868055

**Published:** 2021-01-21

**Authors:** Jessica L. Fraser, Yewande H. Alimi, Jay K. Varma, Tracie Muraya, Tapiwanashe Kujinga, Vanessa K. Carter, Constance Schultsz, Victor J. Del Rio Vilas

**Affiliations:** a Athena Institute, Faculty of Science, Vrije Universiteit Amsterdam, Amsterdam, The Netherlands; b Africa Centres for Disease Control and Prevention, African Union Commission, Addis Ababa, Ethiopia; c US Centers for Disease Control and Prevention, Atlanta, GA, USA; d ReAct Africa, Lusaka, Zambia; e Pan-African Treatment Access Movement, Zimbabwe; f Healthcare Communications and Social Media South Africa, South Africa; g Department of Global Health-Amsterdam Institute for Global Health and Development, Amsterdam UMC, University of Amsterdam, Amsterdam, The Netherlands; h The Centre on Global Health Security, Chatham House, London, UK

**Keywords:** Antimicrobial resistance, Civil Society Organisations, Africa, regional strategy, national action plans

## Abstract

**Background**: Antimicrobial resistance (AMR) is a growing public health threat in Africa. AMR prevention and control requires coordination across multiple sectors of government and civil society partners.

**Objectives**: To assess the current role, needs, and capacities of CSOs working in AMR in Africa.

**Methods**: We conducted an online survey of 35 CSOs working in 37 countries across Africa. The survey asked about priorities for AMR, current AMR-specific activities, monitoring practices, training needs, and preferences for sharing information on AMR. Further data were gathered on the main roles of the organisations, the length of time engaged in and budget spent on AMR-related activities, and their involvement in the development and implementation of National Action Plans (NAPs). Results were assessed against The Africa Centres for Disease Control and Prevention (Africa CDC) Framework for Antimicrobial Resistance (2018–2023).

**Results**: CSOs with AMR-related activities are working in all four areas of Africa CDC’s Framework: improving surveillance, delaying emergence, limiting transmission, and mitigating harm from infections caused by AMR microorganisms. Engagement with the four objectives is mainly through advocacy, followed by accountability and service delivery. There were limited monitoring activities reported by CSOs, with only seven (20%) providing an example metric used to monitor their activities related to AMR, and 27 (80%) CSOs reporting having no AMR-related strategy. Half the CSOs reported engaging with the development and implementation of NAPs; however, only three CSOs are aligning their work with these national strategies.

**Conclusion**: CSOs across Africa are supporting AMR prevention and control, however, there is potential for more engagement. Africa CDC and other government agencies should support the training of CSOs in strategies to control AMR. Tailored training programmes can build knowledge of AMR, capacity for monitoring processes, and facilitate further identification of CSOs’ contribution to the AMR Framework and alignment with NAPs and regional strategies.

## Background

Antimicrobial resistance (AMR) is a complex, broad, and multisectoral health issue that requires strong collaboration and engagement of global, national, and regional stakeholders. Globally, AMR is estimated to cause 700,000 deaths/year [1]. In Africa, the occurrence of AMR is increasing [2–4] with larger impact becoming more apparent [2], with healthcare-associated infections increasing [3]. This growing concern is compounded by weak and fragmented public and animal health systems across the region and a high burden of infectious disease, with 62% of disability-adjusted life years in the Africa region attributable to infectious diseases [3]. A One Health (OH) approach is needed because AMR occurs, and is transmitted, across humans, animals, and the environment, through both direct and indirect contact [4,5].

In May 2015, in response to growing concerns around AMR, the World Health Organization (WHO) developed the Global Action Plan on AMR (GAP) [6]. Member states were urged to develop their own National Action Plans (NAP) by May 2017. However, AMR is not contained by national borders and requires both regional and international oversight. To that effect, regional coordination, alignment of national objectives and activities, and benchmark identification are core activities of the recently launched Framework for AMR 2018–2023 by the Africa Centres for Disease Control and Prevention (Africa CDC) [SM1].

To facilitate the implementation of its Framework, the Africa CDC identified engagement and collaboration with Civil Society Organisations (CSOs) as key. CSOs are non-governmental organisations that operate in the public domain, outside the market (economic structures) and the state (governmental structures) [7], and have a long history of promoting and transforming public health policy and services through mobilising local efforts, providing basic services, innovating service delivery, and advocating for the poorer communities [8]. To date, CSO involvement in AMR-specific activities in Africa has been limited [9]. This is due to the complexity of AMR to explain to diverse audiences, the often-undiagnosed nature of AMR, so thus not at the forefront of patients’ stories, and the limited investment by public health departments into CSOs [9]. This is despite the clear role of CSOs towards AMR efforts [10]. For example, through facilitating awareness campaigns on limiting transmission of AMR and using the expansive reach of CSOs to target local prescribers’ behaviour change [10]. Collaboration of a diverse range of CSOs can help to bind ‘public and private activity together’ [7], needed for an OH approach and support implementation of NAPs through three main activities [11]: service provision, advocacy (communication and community engagement), and enhanced accountability. Service provision includes infection prevention control (IPC), AMR stewardship (appropriate prescription and use of antimicrobials), laboratory diagnosis of AMR (Infection and antimicrobial susceptibility), planning AMR-specific interventions, and reporting. Advocacy and community engagement include education and communication. Accountability activities monitor progress of countries’ efforts toward implementation of NAPs. CSO work on NAPs can directly contribute to implementation of the Africa CDC AMR Framework. To that effect, it is necessary to ensure the alignment of monitoring and evaluation processes and indicators in use by CSOs with those of the NAPs and the Africa CDC Framework. Additionally, for CSOs to have an effective contribution, they must have personnel with a strong level of understanding and knowledge of AMR.

In this manuscript, we surveyed CSOs operating in sub-Saharan Africa with the aim of collating information on their AMR-specific objectives and activities and to understand the specific ways in which CSOs contribute to the region’s efforts towards AMR control. We also assess CSOs capacity-building needs to deliver the above contributions and the preferred mechanisms of information sharing on AMR.

## Methods

In December 2018, the Africa CDC’s Division of Surveillance and Disease Intelligence, held a workshop in Addis Ababa with 37 CSO representatives working in human health, animal health, and the environmental sector to familiarize them with the Africa CDC Framework and identify ways to strengthen CSO activities related to AMR. Workshop attendees formed the basis of the participant sample for this study. We analysed the workshop participant list to identify any significant gaps in country representation and sectors. Efforts to extend the pre-existing list were pursued through a snowball sampling exercise. This method is particularly useful and relevant when the wider population is unknown and extensive [12]; to the best of our knowledge, there is no list of CSOs working in Africa on AMR. Where there were gaps in country or sector representation, a desk search was conducted of possible CSOs engaging in OH and AMR-related work. Four CSOs were identified, by Africa CDC, as key informants (‘champions’) due to their interest and commitment to the aims of the research and their expertise in the topic of AMR. Continuous collaboration with the champions ensured improvement of the research design and the online survey.

The survey response options were designed based on several information sources [11,13], and through an iterative process of discussions with the champions and research team. To design the survey response options to explore the CSO’s activities, the Tripartite questionnaire on global monitoring of country progress on addressing AMR was used as guidance [14]. The Tripartite comprises of the World Health Organization (WHO), the Food and Agriculture Organization of the United Nations (FAO), and the World Organization for Animal Health (OIE). The comprehensive list of activities from the Tripartite survey was adapted to incorporate the three main CSO activities as outlined in by the WHO (2019) [11].

The survey requested the following information: characteristics of CSOs, CSOs AMR priorities, AMR-specific activities, monitoring practices, training needs, and information sharing preferences. CSOs were asked to rank their multiple roles (e.g. advocacy, capacity building) and AMR priorities (e.g. improve surveillance, delay emergence) from a list of options. Types and number of AMR-related activities were classified against the four Africa CDC Framework objectives, namely, improving surveillance, delaying emergence, limiting transmission, and mitigating harm from AMR infections. Further, in each objective, the activities selected by the CSOs were stratified by the sector CSOs self-identified as working primarily on, the sector the activities relate to, and the type of activities (e.g. service provision, advocacy, accountability, or other). Awareness and education activities were separate categories and were assessed against the different levels in the Tripartite survey.^1^^1^Tripartite survey levels for assessment (2018). Level 1 Providing and facilitating activities in parts of the country(ies) to raise awareness about risks of antimicrobial resistance and actions that can be taken to address it Level 2 Providing small-scale antimicrobial resistance awareness campaigns, targeting some but not all relevant stakeholders Level 3 Providing nationwide, government-supported antimicrobial resistance awareness campaigns, targeting all or the majority of relevant stakeholders, based on stakeholder analysis, utilising targeted messaging accordingly within sectors Level 4 Providing targeted, nationwide government-supported activities implemented to change behaviour of key stakeholders within sectors, with monitoring undertaken over the last 2–5 years Responses to training and resource needs were collected through multiple-choice answers, with space for new suggestions. For assessing M&E efforts, CSOs were asked to provide indicators for their main activity in relation to the four Framework objectives. CSO contributions toward developing or implementing NAPs was assessed through yes/no questions. The survey also collected data on the geographic scope of their AMR activities in Africa, length of time working in AMR, and AMR-specific annual budget.

An invitation letter to the online survey was sent to 51 CSOs (37 from the original list, 11 identified through snowballing, and 2 from the desk search). When administering the survey [SM2], online and telephone support was provided to participants at numerous intervals over a two-week period. The questionnaire was in English, and the participants were sent the Africa CDC AMR Framework, along with the survey.

Once the data were collated and analysed, a feedback session was held with the CSO champions to discuss results and establish recommendations.

## Results

### CSO’s sectors and funding

**Figure 1.**
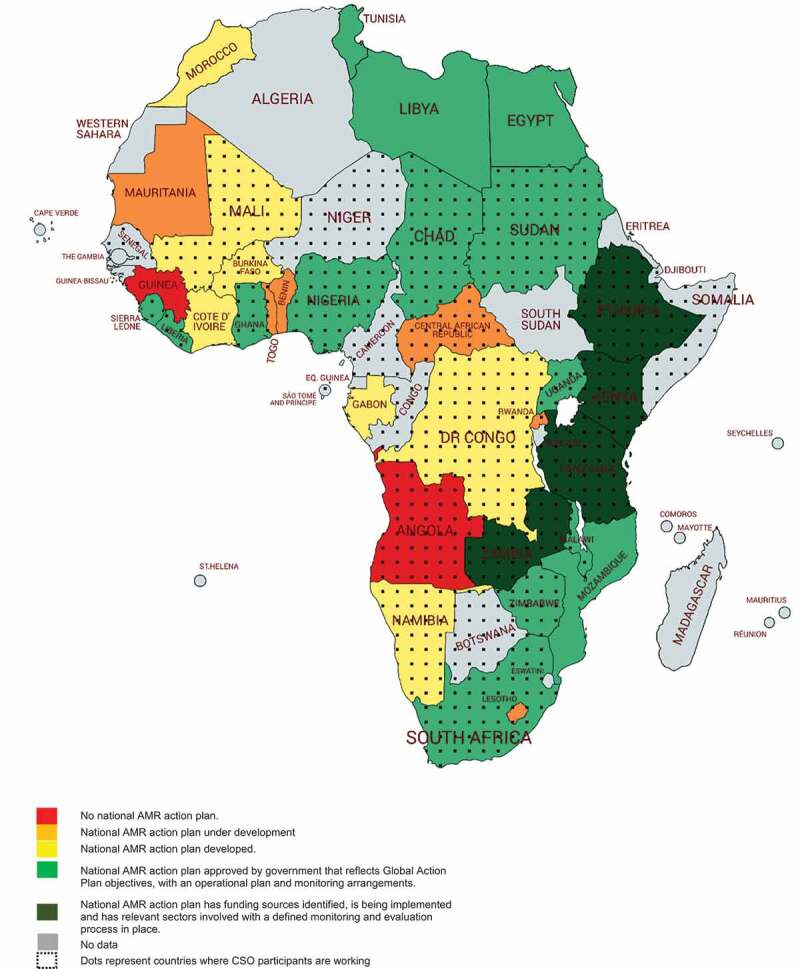
Countries where CSOs are working on AMR in relation to the development of National Action Plans on AMR as reported by the Tripartite database for the period 2018–2019 (14)

The 35 (69%) CSOs that replied to the online survey are engaging in AMR work across 37 countries (out of 54 countries in Africa) (Figure 1). In four countries the NAP has funding sources identified and is being implemented, in 10 countries the NAP has been approved by government, in four countries the NAP is being developed, in two the NAP is under development, one with no NAP in place, and nine where the NAP data are unavailable. No CSOs from northern African countries participated due to limited engagement with the Africa CDC as well as non-response to the survey invitation.

*35 different CSOs completed the survey in some cases represent more than one country. This map does not imply the expression of any opinion whatsoever on the part of Africa CDC concerning the legal status of any country, territory, city or area or of its authorities, or concerning delimitation of its frontiers or boundaries.

Table 1 summarises the characteristics of the CSOs, namely, their main roles, the sector CSOs self-identified as working primarily on, the time they have been working on AMR in Africa, and their involvement in the development or implementation of NAPs. None of the CSOs reported working on plant, food production or food safety sectors. 43% of CSOs delivered advocacy activities, almost one quarter worked in AMR for more than 5 years (although 51% of them worked on AMR for less than 2 years), and about half of them contributed to the development or implementation of the countries’ NAPs.

**Table 1.** CSO profiles by main sector for their operations, main roles, time working on AMR in Africa, and involvement in National Action Plans development and/or implementation

Sectors*	Survey respondents (total 35) (%)
Human	20 (57)
Animal health	10 (29)
Environment	5 (14)
Plant	0
Food production	0
Food safety	0
Main roles**	Count ranking #1*** **(%)**
Advocacy	15 (43)
Service delivery	9 (26)
Technical inputs	5 (14)
Capacity building	3 (9)
Representation	3 (9)
Social functions	1 (3)
Time working on AMR	**Count (%)**
Not started yet	5 (14)
6 months	6 (17)
Less than 1 year	3 (9)
Between one and two years	4 (11)
More than two years	9 (26)
More than five years	8 (23)
CSOs involved in NAP development & implementation	**Count (%)**
Yes development	18 (43)
Human	10 (53)
Animal	6 (33)
Environment	2 (6)
Unknown	3 (9)
Yes implement	18 (51)
Human	12 (34)
Animal	4 (11)
Environment	2 (6)
Unknown	2 (6)

* As the CSOs self-labelled their priority sector.

** Representation (organisations that aggregate citizen voice), advocacy (organisations that lobby on particular issues), technical inputs (organisations that provide information and advice), capacity building (organisations that provide support to other CSOs, including funding), service delivery (organisations that implement development projects or provide services), social functions (organisations that foster collective recreational activities).

*** Ranking was decided by the mean number. Count is the number CSOs ranking the role as #1.

For AMR-related spending, 14 (40%) and four (12%) CSOs invested less than 50K USD and between 51K USD–500K USD in the last fiscal year, respectively. Eleven (31%) CSOs did not report any investment in AMR in the last year, and six (17%) replied that they did not know the exact amount [SM3].

### CSOs AMR priorities

The questionnaire captured current and future CSO priorities. For current priorities, 26 (74%) CSOs chose *limit transmission* of AMR (Africa CDC Objective 3) as first or second priority. *Mitigate harm* (Africa CDC Objective 4) was last. For future priorities, all three sectors prioritised *delay* *emergence* slightly higher than *limit transmission*. When thinking about future priorities, CSOs ranked the objective *improve surveillance* lower, with 37% of the CSOs ranking this objective fourth; in comparison to only 9% of the group ranking the objective *limit transmission* as fourth.

### Type of activities deployed by CSOs

Overall, there is overlap of activities across the different sectors. On the whole, CSOs are engaging predominantly in advocacy-focused activities, followed by accountability-related activities, and service provision [SM4 Graphs 1–4].^2^^2^In the Graphs 1–4 [SM4], comparing the number of activities taking place across the sectors needs to be taken with caution as there were more CSOs working in the human sector – human sector represented by 20 CSOs, animal sector by 10 CSOs, environmental sector by 5 CSOs. Instead, the number of activities (sizes of bubbles) within the same sector can be compared. One limitation is that the overlap of activity choices, in the survey, for animal and food production reduces the ability to distinguish the CSOs’ specific focus. Most CSOs (n = 26, 74%) are engaging in awareness raising activities only in parts of the country (ies) they operate, and not yet nation-wide. The activity ‘providing tailored ad hoc AMR training courses for a reduced number of human health workers, or with mostly a local coverage’ was chosen by 12 (32%) CSOs.

### Monitoring and evaluation

There were only 20 examples (14% of a possible 140^3^^3^If all 35 CSOs were to provide a metric for all four objectives.) of monitoring metrics. CSOs working in human health provided the most metrics. One example provided, under the objective *delay emergence*, was monitoring the number of legislation pieces, guidelines, or regulations developed. Another example, under the objective *limit transmission*, was monitoring the number of training events on good productions practices. Participants did not provide further explanation of the metrics used. Only eight CSOs (23%) reported having an AMR strategy for Africa, and 97% responded that this strategy was not publicly available. However, 51% of the CSOs claimed to systematically monitor most of their activities against their own strategy. Out of the 18 CSOs who are helping to implement a NAP, only three specified using an overarching framework like a NAP for monitoring their activities [SM5].

### Necessary resources and trainings

The most commonly requested resource by CSOs was ‘training in basic principles of AMR’. This and other training needs are shown in Table 2 CSOs expressed the need for technical skills in data analysis and dissemination techniques for effective advocacy campaigns.

**Table 2.** Types of capacity building support needed by CSOs to deploy AMR-related activities

Resources	# of times selected*	% of CSO choosing this resource (35) *
Training in basic principles of AMR	21	60
Access to best practice examples/case studies	19	54
Networking opportunities	17	49
Planning tools (such as stakeholder analysis)	14	40
Information on national and global AMR policies and plans	14	40
Targeting tools (such as websites, blogs, and media engagement)	13	37
Monitoring and evaluation tools (such as Outcome Mapping)	13	37
Access to data and scientific reports	12	34
Technical guidance	7	20
Other	6	2
Training topic	**# of times ranked as choice 1**	**% of CSOs #1 choice (35)**
Understand the development and main causes of AMR	10	29
Training in communication to influence behaviour change in all relevant sectors	8	23
Understand the basic principles of infection prevention and control (IPC), i.e. hand hygiene to prevent transmission of infections and health-associated infections (HAIs)	5	14
Training in communication to empower health-care providers to challenge misuse or overuse of antibiotics	3	9
Understand the potential for cost savings and health gains associated with effective infection control and appropriate antimicrobial use	2	6
Understand the impact of resistance on choice of antimicrobial therapy for treating infections	1	3
Understand the morbidity, mortality and economic threat of AMR to human health	1	3
Understand antimicrobial use in food-animal production. Problems, solutions, challenges	1	3
Understand local AMR epidemiology, resistance and susceptibility patterns and use of guidelines	1	3
Understand the diagnostic role of the microbiology laboratory in detecting infections, resistance patterns, guiding patient management and informing AMR control strategies	1	3
Training in digital skills and online-advocacy methods	1	3
Training in skills such as: communication of public messages, monitoring and evaluation, proposal writing	1	3

* In several cases the CSOs chose more than one option. For this reason, the sum of votes is above 35.

### Information sharing

CSOs replied that they receive most of their information about AMR from other CSOs, and not from local authorities. Animal-focused CSOs also receive information from private companies. Across all sectors, email is the most commonly used platform to receive information about AMR, followed by conferences attendance. Virtual online meetings are the least used platform for sharing information. For dissemination of AMR information, social media was the preferred method, followed by conference presentations and website posts [SM6]. CSOs also provided a number of suggestions for further engagement: use of WhatsApp, information sharing in places of worship (for example mosques, temples, churches), and the need to reduce duplication and facilitate coordination across regional, national, and global AMR platforms.

## Discussion

To the best of our knowledge, ours is the first effort to describe civil society work on AMR control in Africa. CSOs are active in every objective as listed by Africa CDC AMR Framework, and activities are taking place across all sectors. The type of activity engaged most with is advocacy. There appears to be opportunities for more robust M&E, and there is a need for training in basic knowledge of AMR. CSOs communicate with and receive most information about AMR from other CSOs.

The diverse group of CSOs are engaging in activities across all sectors, demonstrating a One Health approach to AMR [5]. The activities outlined in the survey highlight where CSOs are overlapping in their aims and activities, and where points of collaboration exist. Cross-sector activities also provide tangible entry points for new CSOs to become engaged in activities outside of their primary sector. For awareness-building, activities at both regional and national level were identified. This provides entry points for ‘collaborative, multi-sectoral, and transdisciplinary efforts at the local, regional and national level’ [15]. Additional entry points can be explored by using the communication channels identified in the survey. For example, the animal CSOs have communication channels with both the private and public sector. These CSOs can have an important role in binding ‘public and private activity together’ [7] by being facilitators of AMR-related information.

Mitigate harm was placed as the last priority by the CSOs, in every sector. However, the number of activities reported by CSOs in this objective was higher than the other three. This suggests that there may be some confusion around the AMR Framework objectives. Improving clarity of the Framework objectives, and making them more relatable to the CSOs’ work and focus, can help CSOs to plan with the Framework in mind, and can become more engaged in AMR-related work by understanding their role and current and potential contribution to the Framework. There is no one single objective that CSOs are contributing to most; rather, this exemplifies the extensive and diverse role CSOs can play in the Framework implementation.

Advocacy is the dominant type of activity by CSOs. To advocate affectively, CSOs expressed the need to enhance their understanding of the basics of AMR and data analysis to ensure messages can be clear and comprehensible and use of evidence is appropriate; especially when sharing information amongst fellow CSOs. The lack of this basic knowledge may limit the CSOs ability or confidence to lead on AMR advocacy and awareness campaigns. Furthermore, if CSOs are to play a ‘crucial role as a vehicle for communicating for public awareness and behaviour change’ [10], then CSOs need the capacity and skills to communicate effectively. Further efforts are needed to identify how advocacy activities can make the message of AMR more understandable, and how the Africa CDC can support CSOs to monitor the impact of their advocacy efforts. Further research can also seek to identify which specific bodies (such as Ministries of Health) CSOs are targeting with their accountability activities. This can enable more specific monitoring metrics to be established.

CSOs were rarely involved in delivering services. This is interesting as CSOs, especially in the context of Africa, are recognised as being one of the main providers of health services – with 70% of healthcare provision coming from CSOs [7]. A possible explanation for this lack of engagement in AMR-specific service delivery activities may stem from CSOs’ only recent involvement in AMR (Table 2); service delivery is further along in the implementation of AMR-related activities and it requires greater operational capability, as opposed to planning and advocacy. Nonetheless, service delivery appears as an important type of activity in the implementation of the Africa CDC Framework, especially as they include IPC practices [6]. Examples of service delivery activities, such as ‘*providing activities to develop and promote good management and hygiene practices’*, which have been identified by CSOs in this survey, can be explored further to asses if they can be scaled-up or if there are opportunities for other CSOs to incorporate these service-delivery activities into their current remit. This must be done in collaboration with local authorities to ensure alignment with NAPs and aid the capacity of CSOs to implement these activities. The activity *‘mobilising support for the objective to be on the governments’ agenda’*, was one of the main activities chosen by the CSOs for all four objectives in every sector. This shows how CSOs are contributing to tackling AMR at a macro level, through influencing national policy on AMR.

Monitoring frameworks are needed to track, assess, and report on the activities taking place towards implementing NAPs. Our results show that 51% of the CSOs have strategies to monitor their general activities; however, only 23% have a specific AMR-focused strategy. This is not surprising as many CSOs have only recently started work in AMR, with 51% working in AMR for less than two years and 40% have spent less than 50K USD on AMR-related activities. Limited M&E was also reported for global One Health networks [16]. CSOs strategies need to be explored, and related back to the Africa CDC Framework and NAP, to see if activities are already incorporated in the strategy and efforts can be aligned and coordinated to improve efficiency [16]. Monitoring processes ensure that the essential activities by CSOs are executed. The nature of CSOs as non-profit and non-governmental organisations means that responsibility, accountability, and transparency of these activities, is fundamental to their success and good performance, and the creation of a competitive edge amongst other CSOs [16]. Reporting of monitoring processes and metrics, on the CSO’s own activities and their outputs and outcomes, is necessary for CSOs’ to ensure credibility and trust amongst development programmes [16], and thus, increase potential for securing investment and support. Furthermore, effective monitoring systems can produce data that when highlighted by CSOs could generate policy triggers [10]; for example, monitoring of resistant pathogens found on retail shelves can aid to alert the public and regulatory authorities [10], and initiate action for relevant regulatory authorities. Policy triggers can help to mobilise government action, and resources, to support progress towards GAP outcomes.

We cannot claim that our sample of CSOs is representative of the actual population of CSOs working on AMR in Africa, across the different sectors. We did not manage to engage with CSOs operating in northern African countries, although we did identify CSOs working in these countries. Strategies to engage northern countries need to be explored, e.g. the translation of communications around the research, and the survey itself, to French or Arabic might encourage interest from northern-African countries. Further, we recommend that a comprehensive stakeholder mapping analysis is carried out to illustrate the CSOs currently working on AMR, and those with the potential to, across the continent.

### Perspectives and strategic fit

The survey provides a relevant tool for future exploration of CSOs contribution to AMR control and their training needs. As this was the first survey to CSOs, our findings necessarily inform generic processes in CSOs’ contribution to AMR control. For example, the actual contribution by CSOs to NAPs could not be established from this survey alone. Future surveys can be used to re-assess if, and how, priorities, monitoring practices, and training needs have changed, and what are the current barriers for CSOs in improving knowledge on AMR. Future surveys should focus on specific types of CSOs and activities of specific interest to CSOs to attain more specific data. Surveys should also incorporate additional activity options and should explore monitoring metrics more extensively. Building upon the preferred information sharing platform expressed by the CSOs, results from the survey can be shared via healthcaresocial media/blog posts. This method can enable wider distribution of results. Stemming from this survey engagement, the first cohort of CSOs were selected and trained on the following: basics of AMR, IPC, OH and communication and advocacy. In February 2020, the CSO network attended a Capacity Building and Workforce Development Workshop. At this, CSOs committed to work together, share best practices, and promote synergies in the priority actions.

The significance of civil society in Africa came in the 1990s as a result of democratization across the world, economic decline, privatisation, and increase in donor aid [17]. In the 1980s, many areas of the world were experiencing a shift to ‘democratization’ [17] whereby the focus was moving from the state to the potential capacity for societal institutions to mobilise democratization [17]. Economic ‘mismanagement’ across the African continent in the 1980s fuelled the ‘disengagement from the formal economy’ [17] due to illegal activities and gave rise to pro-democracy movements by way of civil society institutions. The concerns regarding the reliable execution of ‘good governance’ [17] prompted donor objectives, and support, to instead be directed towards civil society organisations. The foremost experiences of this shift provided successful examples, such as South Africa, and paved the way for increased support for CSOs in other areas of Africa. The increased emphasis on privatisation by donor aid policy prompted more support and direction to be placed on CSOs providing public services, especially in healthcare. Now, international CSOs are abundant across Africa; and are a crucial pillar in healthcare systems. This was particularly exemplified in the HIV/AIDS response where CSOs are viewed as playing a ‘exceptional’ [9] role in the global response.

However, as more emphasis and pressure has been placed on CSOs to provide public services, CSOs (including faith-based organisations) now have to rely on a ‘combination of government resources, user fees from patients, development assistance from bilateral and multilateral donors, and funding and in-kind contributions from within-country faith groups and local communities’ [18]. This fragmented nature of funding streams means that CSOs have different levels of resources, capacity, and thematic focus to deliver health services, advocacy, and accountability activities. As well as there is no formal tracking of these fragmented funding streams for CSOs [18].

It is recognised that main obstacles for CSOs in engaging in priority setting and policy making are the following: limited capacity, limited funding, and CSOs are not recognised for their importance and relevance in healthcare decision-making [8]. Furthermore, CSOs are often in competition for funding and influence, and ‘too often appear to live in parallel universes and do not engage across boundaries’ [8]. Therefore, CSOs are often not benefiting from the possible support, funding, political influence, and capacity building that are within networks [8].

However, CSOs have indeed been recognised for their important role in the control of AMR. The Global Action Plan (2015) [6] outlines that CSOs’ roles should ‘help to promote public awareness and understanding of infection prevention and use of antimicrobial medicines across all sectors’.

Additionally, the Antibiotic Resistance Coalition (ARC) explicates that, ‘civil society should be recognized and included for its critical role as a vehicle for communicating for public awareness and behaviour change over AMR, and this should be an integral part of the implementation of National Action Plans’ [10].

Through our research, and in consideration of the above literature on AMR and CSOs, we aim to highlight that in order for CSOs’ efforts in AMR control to be prioritised and sufficiently funded, the following practical recommendations need to be addressed and further explored:

- By evidencing and highlighting their relevance and implementation of NAPs, CSOs’ work and role can be recognised by the AMR Technical Working Groups for each country’s NAP. Through recognising the contribution that CSOs are currently making, and potentially could make, to the NAP, CSOs should be included in Technical Working Group meetings.
- CSOs engage in more collaboration instead of competition against each other. This can be facilitated and promoted through the AMR CSO network, which the Africa CDC is providing training and workshops to.
- The complex problem of AMR requires a One Health (OH) approach. This is because AMR exists and is transmitted through numerous interfaces across humans, animals, and the environment through direct and indirect contact [5]. We have seen that CSOs are working in a OH approach due to their crossover in activities. The Africa CDC and the respective countries’ Technical Working Groups for AMR can explore these crossovers further and support communication between organisations.

## Conclusion

Our results offer some insight into the landscape of CSOs currently engaged in AMR control efforts in Africa. CSOs from across different sectors are contributing to all four Africa CDC AMR Framework objectives. CSOs are focusing on advocacy activities to promote AMR control; however, they require additional knowledge and training to effectively communicate the complexity of the problem. Tailored training programmes can build knowledge and capacity for monitoring processes and facilitate further contribution by CSOs to the AMR Framework and NAPs. A network of engaged CSOs has been established, now the focus is to ensure AMR information is shared accurately and effectively across the network and beyond.

## Supplementary Material

Supplemental Material

## Paper context

This research was conducted to assess and highlight the important role that CSOs can play in controlling and preventing of antimicrobial resistance. To our knowledge, there is no recognised approach to explore and monitor the role of CSOs in the global action on AMR. This study aims to contribute to that aim.
