# Supplementary material for: Antimicrobial resistance control efforts in Africa: a survey of the role of Civil Society Organisations
Source: Glob Health Action. 2021 Jan 21;14(1):1868055. doi: 10.1080/16549716.2020.1868055 (PMC7833050; doi:10.1080/16549716.2020.1868055)
Supplement: Supplemental Material [file ZGHA_A_1868055_SM7056.docx]

**Supplementary material**

### SM1: Africa CDC Framework for Antimicrobial Resistance Control

Table 4. Priority areas for Africa Centres for Disease Control and Prevention framework for antimicrobial resistance control, 2018–2023 (20)

| Strategic plan area | Activities |
| --- | --- |
| IMPROVE SURVEILLANCE | Increase the number of tests performed on humans and animals for AMR organisms  Increase the proportion of clinical diagnostic laboratories with quality assurance programmes  Increase the proportion of public health laboratories with quality assurance programmes and international accreditation  Increase the number of national public health laboratories conducting surveillance for AMR using standardised protocols  Increase the number of member states that continuously collect, analyse, report, and disseminate data about AMR for high priority pathogens in their respective countries |
| Delay emergence | Increase the proportion of physicians adhering to prudent antibiotic use guidelines  Increase the proportion of veterinarians and food producers adhering to prudent antibiotic use guidelines  Reduce availability and sales of substandard and counterfeit antibiotics |
| Limit transmission | Increase the proportion of health care facilities implementing infection control and prevention programmes  Increase the availability and sales of animal products raised with prudent antibiotic use |
| Mitigate harm | Increase the number of health care facilities with quality diagnostic tests for infection and AMR  Reduce the availability and use of substandard diagnostic tests and supplies  Increase the proportion of physicians and health care facilities adhering to guidelines for treatment of susceptible and AMR infections in humans  Maintain access to essential antibiotics |
| cross-cutting | Advocate for policies and laws to enable long-term prevention and control of AMR  Civil society engagement  Develop human resources for AMR surveillance and control |

### SM2: Full Qualtrics Survey and Informed consent

Antimicrobial Resistance CSO Survey - 2019

**Start of Block: Introduction**

Q1 **Welcome to the Civil Society Organisation Antimicrobial Resistance Survey**

This survey is a collaboration between the Africa Center for Disease Control and Prevention (Africa CDC) and Civil Society Organisations (CSOs) towards the Antimicrobial Resistance (AMR) Framework implementation

The aim of this survey is to gain further understanding of the needs of CSOs and how specifically Africa CDC can help CSOs to implement the Africa CDC AMR Framework, with the overall aim to work together to advance AMR control in Africa.

In this survey, we would like to:

- Understand how the CSOs can contribute to the AMR Framework
- Identify the specific training topics and needs of CSOs

The information gathered will directly inform the types of training modules to be offered to CSOs and the ways in which the Africa CDC can support such training; as well as to provide support towards activities, resource allocation, and development.

As well as to gain a better understanding of the current AMR-focused activities amongst CSOs.

This survey is made up of 6 sections and will take approximately 30 minutes. **You can save and come back to your answers at different times throughout the survey period.**

Please complete the whole survey by **6pm [Central African Time] on Friday, May 31st.**

If you have any problems with completing the survey or have any questions throughout the survey, please do not hesitate our researcher, Jessica Fraser, at the email: jessicafraser02@gmail.com

Thank you very much for your willingness to participate in this survey. Your input is important to controlling AMR in Africa as well as for the long-term partnership between CSOs and the Africa CDC.

With kind regards on behalf of the Africa CDC, 
 Jessica Fraser

 Jay K. Varma, MD
 Senior Advisor, Africa CDC

| Page Break |  |
| --- | --- |

Q2 **Informed consent**
 
Before starting the survey, we want to inform you of your consent for the survey.

Your participation in this study is voluntary. You can choose not to participate and can withdraw from the study at any time. The results of the research study may be used for a report or scientific paper, but your name and your organisation's name, and details, will be kept anonymous. 

The data gathered will be analysed and used to inform the activities of the Africa CDC. The survey will be analysed and presented aggregated, and no individual organisation will be identified. 
 
By submitting this consent, you are indicating that you have read the description of the study and that you agree to the terms as described.
 
If you have any questions about this informed consent before starting the survey, please email Jessica Fraser at: jessicafraser02@gmail.com 
 
Please click the box below to confirm your consent. We greatly appreciate your participant.

- Yes, I give my consent
- No, I have a question before starting the survey

*Skip To: Q3 If Informed consent      Before starting the survey, we want to inform you of your consent for the s... = No, I have a question before starting the survey*

*Display This Question:*

*If Informed consent      Before starting the survey, we want to inform you of your consent for the s... = No, I have a question before starting the survey*

Q3 Please email Jessica at jessicafraser02@gmail.com with your question before participating in the survey.

*Skip To: End of Survey If Please email Jessica at jessicafraser02@gmail.com with your question before participating in the...() Is Displayed*

| Page Break |  |
| --- | --- |

**End of Block: Introduction**

**Start of Block: Section 1**

*Display This Question:*

*If Informed consent      Before starting the survey, we want to inform you of your consent for the s... = Yes, I give my consent*

Q4
Section 1: Background 

What is the name of your organisation? [This information will not be shared outside of the research team]

________________________________________________________________

| 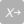 |
| --- |

Q5 In what country (ies) does your organisation deliver or deploy AMR work? 

[To select more than one, hold down the Ctrl key on your keypad and click the country names]

[Drop down list of all countries in Africa]

| 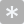 |
| --- |

Q6 Please rank the sectors in which your organisation deploy AMR work.

[Please rank the sectors from 1- 7, with **1 being the main sector of your operations**, and 7 being the least. **If you do not use the 'Other' please write 'NA' and number it as '7**']

______ Human health

______ Animal health (terrestrial and aquatic)

______ Environment (including water, sanitation and hygiene (WASH))

______ Plant health

______ Food production

______ Food safety

______ Other (please specify)

| 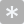 |
| --- |

Q7 In regards to AMR, what are the main activities of your organisation?
 
[Please rank these from 1 to 6, with 1 being main activity, and 6 being the least]

______ Representation (organisations that aggregate citizen voice)

______ Advocacy (organisations that lobby on particular issues)

______ Technical inputs (organisations that provide information and advice)

______ Capacity building (organisations that provide support, including funding)

______ Service delivery (organisations that implement development projects or provide services)

______ Social functions (organisations that foster collective recreational activities; such as: cultural groups, choral groups, sports clubs, prayer or mosque groups)

**End of Block: Section 1**

**Start of Block: Section 2: Development of National Action Plans**

Q8 **Section 2: Development of National Action Plans for AMR**
 
This section will include 3 short questions related to your organisation's involvement in National Action Plans for AMR.

Q9 Has your organisation contributed (or is currently contributing) to the **development** of national action plans (NAPs) for AMR?  Please note that our focus here in NAP development.

- Yes
- No
- I don't know

Q10 Has your organisation contributed (or is currently contributing) to the **implementation** of national action plans (NAPs) for AMR? Please note that our focus here in NAP implementation.

- Yes
- No
- I don't know

Q11 Was your organisation involved in the country(ies) multi-sectoral group that replied to the Tripartite Country Monitoring Progress questionnaire on AMR (2018)? 
The 'Triparite' includes the World Health Organisation (WHO), the Food and Agriculture Organisation (FAO), and the World Organisation for Animal Health (OIE), who are taking collective action to minimise the emergence and spread of AMR.

- Yes
- No
- I don't know

**End of Block: Section 2: Development of National Action Plans**

**Start of Block: Section 3: OBJECTIVES: Africa CDC AMR Framework**

Q12 Section 3: AMR Objectives 
 
This section will ask questions related to your organisation's interest and work towards the objectives included in the Africa CDC Framework for AMR. 
CSOs which have recently started working towards AMR may not have all the information to answer the questions or may not have started working towards every objective. Please kindly answer as many as you can to your knowledge and when information is not known, please select 'I don't know'.

| 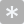 |
| --- |

Q13 The Africa CDC AMR Framework outlines four main priority areas for the next five years: 

- **Improve surveillance of AMR organisms among humans and animals** (Strengthening laboratory capacity for surveillance in humans and animals)
- **Delay emergence of AMR** (Reducing the need for and inappropriate use of antimicrobials, and by promoting prudent antimicrobial use in collaboration with animal health, agriculture, and other related authorities, can help to delay emergence of AMR)
- **Limit transmission of AMR** (Limiting the transmission of AMR in healthcare facilities through infection control and prevention activities, and in animals through good agricultural practices)
- **Mitigate harm among patients infected with AMR organisms** (Reducing potential harm from AMR by promoting antimicrobial and diagnostic stewardship, while seeking ways to maintain access to lifesaving diagnostics and treatment)

**Q**: Looking at the objectives of the Africa CDC Framework for controlling AMR, which objective or objectives does your organisation focus its activities on?  

[Please rank the four options from 1-4, with 1 being the objective you are working towards the most, and 4 being the least]

______ Improve surveillance of AMR organisms among humans and animals

______ Delay emergence of AMR

______ Limit transmission of AMR

______ Mitigate harm among patients infected with AMR organisms

| 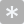 |
| --- |

Q14 Which of the four objectives are you interested in focusing on in the future? 


[Please rank the four options from 1-4, with 1 being the most interested, to 4 being the least interested]

______ Improve surveillance of AMR organisms among humans and animals

______ Delay emergence of AMR

______ Limit transmission of AMR

______ Mitigate harm among patients infected with AMR organisms

Q15 When did your organisation start working in the topic of AMR in Africa?

- In the last 6 months
- In the last year
- Between one and two years ago
- More than two years ago
- More than five years ago
- Not started yet

Q16 What was the level of investment or cost of your operations against AMR **in your last financial year** (in US $)?  [Please include all direct and indirect costs] 


['Investing' means any funding spent towards AMR-related activities, human personnel, technology]

- Less than $50,000
- Between $51,000 - $100,000
- Between $101,000 - $200,000
- Between $201,000 - $500,000
- More than $500,000
- Nothing invested on AMR
- I don't know

Q17 What was the level of investment or cost of your operations against AMR on **average in the last 5 years**(in US $)?  [Please include all direct and indirect costs] 

['Investing' means any funding spent towards AMR-related activities, human personnel, technology etc...]

- Less than $50,000
- Between $51,000 - $100,000
- Between $101,000 - $200,000
- Between $201,000 - $500,000
- More than $500,000
- Nothing invested on AMR
- I don't know

Q18 Has your organisation increased its investment on AMR in the last 5 years?

- Yes
- No
- I don't know

**End of Block: Section 3: OBJECTIVES: Africa CDC AMR Framework**

**Start of Block: Section 4: ACTIVITIES**

Q19 **Section 4: AMR Activities**
 
This section includes questions to cover the types of activities that your organisation is doing towards general awareness of AMR, education on AMR, and activities towards the four Africa CDC AMR Framework objectives. 
 
CSOs that have recently started to develop their response to AMR may not be engaging in activities for every objective or for every sector (for example, your organisation may not be involved in food production). Therefore, the response 'None of these' or 'I don't know' is appropriate to use. 

If your organisation is carrying out activities that are not represented in these answer options, please provide examples of your activities in the open box. This information is helpful towards better understanding the activities of CSOs. 

The last two questions ask about monitoring of activities. For these, it is also possible to state 'No activity' or 'I don't know' if this information is unknown by your organisation.

Q20 What activities is your organisation doing in relation to **raising general awareness of AMR**?

[Please choose **only one option** that best describes the scale of your AMR awareness activities **OR** provide an activity in the 'Other activities' box]

- Providing and facilitating activities in parts of the country(ies) to raise awareness about risks of antimicrobial resistance and actions that can be taken to address it
- Providing small-scale antimicrobial resistance awareness campaigns, targeting some but not all relevant stakeholders
- Providing nationwide, government-supported antimicrobial resistance awareness campaigns, targeting all or the majority of relevant stakeholders, based on stakeholder analysis, utilising targeted messaging accordingly within sectors
- Providing targeted, nationwide government-supported activities implemented to change behaviour of key stakeholders within sectors, with monitoring undertaken over the last 2-5 years
- Other activities (please specify) ________________________________________________
- None of these
- I don't know

| 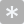 |
| --- |

Q21 What activities is your organisation doing in relation to **education** on AMR and**HUMAN HEALTH:**

[Please choose **only one option** that best describes the scale of your AMR education activities **OR** provide an activity in the 'Other activities' box]

- Providing tailored ad hoc AMR training courses for a reduced number of human health workers, or with mostly a local coverage
- Providing tailored ad hoc AMR training courses for most human health workers, or with a nationwide coverage
- Other activities (please specify) ________________________________________________
- None of these
- I don't know

| 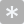 |
| --- |

Q22 What activities is your organisation doing in relation to **education** on AMR and **ANIMAL HEALTH:**
 
[Please choose **only one option** that best describes the scale of your AMR education activities **OR** provide an activity in the 'Other activities' box]

- Providing tailored ad hoc AMR training courses on antimicrobial resistance and antimicrobial use for a reduced number of veterinary related professionals, or with mostly a local coverage
- Providing tailored ad hoc AMR training courses on antimicrobial resistance and antimicrobial use is available nationwide for veterinary related professionals
- Other activities (please specify) ________________________________________________
- None of these
- I don't know

| 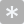 |
| --- |

Q23 What activities is your organisation doing in relation to **education** on AMR and the **ENVIRONMENT and FOOD PRODUCTION**:
 
[Please choose **only one option** that best describes the scale of your AMR education activities **OR** provide an activity in the 'Other activities' box]

- Providing tailored ad hoc AMR training courses for a reduced number of stakeholders, or with mostly a local coverage
- Providing tailored ad hoc AMR training courses for most stakeholders, or with a nationwide coverage
- Other activities (please specify) ________________________________________________
- None of these
- I don't know

| Page Break |  |
| --- | --- |

Q24 **Questions related to types of activities towards the four Africa CDC AMR Framework objectives**

[For these questions, please tick **as many activities that are relevant** to your organisation.  Please also feel free to **add any additional activities**]

**Objective 1: For improving surveillance of AMR organisms among humans and animals**: What activities is your organisation doing towards this?

**In HUMAN health:**

- Providing laboratories, and/or reagents and other commodities, for surveillance
- Campaigning for prescribing practices and appropriate antibiotic use to be monitored in healthcare settings
- Scale-up the proportion of quality public health laboratories with quality assurance programmes
- Ensuring that on a regular basis (every year/two years) data is collected and reported on: a) Antimicrobial sales or consumption at national level for human use; and b) Antibiotic prescribing and appropriate/rational use, in a representative sample of health facilities, both public and private (4)
- Encouraging governments to scale up their reporting and dissemination of data on AMR for high priority pathogens
- Mobilising support for this objective to stay on governments' agenda (6)
- Other activities (please specify) ________________________________________________
- None of these
- I don't know

Q25 **Objective 1: For improving surveillance of AMR organisms among humans and animals**: What activities is your organisation doing towards this?

**In ANIMAL health:**

- Ensuring there is a plan agreed for monitoring quantities of antimicrobials sold for/used in animals, based on The World Organisation for Animal Health (OIE) standards
- Campaigning that data to be collected and reported on the total quantity of antimicrobials sold for/used in animals and their intended type of use (therapeutic or growth promotion).
- Scale-up the proportion of quality clinical diagnostic laboratories with quality assurance programmes
- Ensuring that on a regular basis, data is collected and reported to the National Ministry in charge of Veterinary services and/or OIE on the total quantity of antimicrobials sold for/used in animals nationally, by antimicrobial class, by species (aquatic or terrestrial), method of administration, and by type of use (therapeutic or growth promotion)
- Ensure that data on antimicrobials used under veterinary supervision in animals are available at farm level, for individual animal species
- Mobilising support for this objective to stay on governments' agenda
- Other activities (please specify) ________________________________________________
- None of these
- I don't know

Q26 **Objective 1: For improving surveillance of AMR organisms among humans and animals**: What activities is your organisation doing towards this?

**In the ENVIRONMENT and FOOD PRODUCTION**& FOOD SAFETY**:**

- Ensuring there is a plan agreed for monitoring quantities of pesticides used for the purpose of controlling bacteria or fungal diseases
- Campaigning for data to be collected and reported on for total quantity of pesticides sold and is used nationally for the purpose of controlling bacteria or fungal diseases
- Scale-up the proportion of laboratories that follow quality assurance processes in relation to foodborne pathogens and/or relevant indicator bacteria
- Ensuring that on a regular basis, data is collected and reported on the quantity of pesticides sold/used in plant production for the purpose of controlling bacteria or fungal diseases
- Monitoring the levels of antimicrobials in water bodies, soil etc.
- Mobilising support for this objective to stay on governments' agenda
- Other activities (please specify) ________________________________________________
- None of these
- I don't know

| Page Break |  |
| --- | --- |

Q27 **Objective 2: For delaying emergence of AMR:** What activities is your organisation doing towards this?

**In HUMAN HEALTH:**

[For these questions, please tick as many activities that are relevant to your organisation.  Please also feel free to add any additional activities]

- Promoting the implementation of national policies for antimicrobial governance for the community and health care settings
- Promoting practices for appropriate antimicrobial use in healthcare facilities and ensuring guidelines for appropriate use of antimicrobials are available
- Monitoring if guidelines and other practices are implemented in most health facilities nationwide
- Using surveillance results to inform and scale-up government action and to update treatment guidelines and essential medicines lists
- Ensuring data on use of antibiotics is systematically reported and fed back to prescribers
- Mobilising support for this objective to stay on governments' agenda
- Other activities (please specify) ________________________________________________
- None of these
- I don't know

Q28 **Objective 2: For delaying emergence of AMR**: What activities is your organisation doing towards this?

**In ANIMAL HEALTH:**

- Promoting national legislation to cover all aspects of national manufacture, import, marketing authorization, control of safety, quality and efficacy and distribution of antimicrobial products for food animals (1)
- Ensuring the national regulatory framework for AM products incorporates all the elements included in the related international standards on responsible and prudent use of antimicrobials (e.g. OIE Terrestrial and Aquatic Codes, Codex Alimentarius) according to animal species and/or production sector
- Monitoring if veterinarians and food producers are adhering to prudent antibiotic use guidelines
- Checking enforcement processes and control are in place to ensure compliance with legislation
- Campaign against the sales of substandard and counterfeit antibiotics
- Mobilising support for this objective to stay on governments' agenda
- Other activities (please specify) ________________________________________________
- None of these
- I don't know

| Page Break |  |
| --- | --- |

Q29 **Objective 3: For limiting transmission of AMR**: What activities is your organisation doing towards this?

**In HUMAN HEALTH:** 

[For these questions, please tick as many activities that are relevant to your organisation.  Please also feel free to add any additional activities]

- Promoting and ensuring a national infection prevention control (IPC) programme or operational plan is available. *National IPC and water, sanitation and hygiene (WASH) and environmental health standards exist but may not be fully implemented*
- Promoting and implementing a national IPC programme and operational plan and national guidelines for health care IPC. *Selected health facilities are implementing the guidelines, with monitoring and feedback in place.*
- Ensuring a national IPC programme is available and IPC plans and guidelines implemented nationwide. *All health care facilities have a functional built environment (including water and sanitation), and necessary materials and equipment to perform IPC, per national standards*
- Ensuring compliance and effectiveness are regularly evaluated and published. *Plans and guidance are updated in response to monitoring results*
- Encouraging government to scale up the proportion of health care facilities implementing infection control and prevention programmes
- Mobilising support for this objective to stay on governments' agenda
- Other activities (please specify) ________________________________________________
- None of these
- I don't know

Q30 **Objective 3: For limiting transmission of AMR:** What activities is your organisation doing towards this?

**In ANIMAL HEALTH:**

- Providing activities to develop and promote good production practices; such as: the availability and sales of animal products raised with prudent antibiotic use
- Involved in developing the national plan to ensure good production practices in line with international standards (e.g. OIE Terrestrial and Aquatic Codes, Codex Alimentarius). Nationally agreed guidance for good production practices developed, adapted for implementation at local farm and food production level
- Promoting a nationwide implementation of the plan to ensure good production practices and national guidance is published and disseminated
- Ensuring the plan is monitored for the impact on level of AMR, on animal health and welfare, and on production
- Updating the plans and guidance in response to findings from monitoring outcomes
- Mobilising support for this objective to stay on governments' agenda
- Other activities (please specify) ________________________________________________
- None of these
- I don't know

Q31 **Objective 3: For limiting transmission of AMR:** What activities is your organisation doing towards this?

**In the ENVIRONMENT and FOOD PRODUCTION & FOOD SAFETY:**

- Providing activities to develop and promote good management and hygiene practices
- Helping to develop the national plan to ensure good management and hygiene practices in line with international standards (e.g. Codex Alimentarius)
- Ensuring guidance for good practices is agreed, and adapted for implementation according to local food processing approaches
- Monitoring the plan for the impact on level of AMR, on animal health and welfare, and on production
- Providing services to implement the plan nationwide and ensuring national guidance is published and disseminated
- Mobilising support for this objective to stay on governments' agenda
- Other activities (please specify) ________________________________________________
- None of these
- I don't know

| Page Break |  |
| --- | --- |

Q32 **Objective 4: For mitigating harm among patients and/or animals infected with AMR organisms:** What activities is your organisation doing towards this?

**In HUMAN HEALTH:**

[For these questions, please tick as many activities that are relevant to your organisation.  Please also feel free to add any additional activities]

- Encouraging governments to enforce a national policy for antimicrobial governance and regulation, that addresses appropriate use, availability and quality of antibiotics in the community and in health care settings
- Campaigning to maintain access to essential antibiotics
- Promoting practices to assure appropriate antimicrobial use is being implemented in healthcare facilities and guidelines for appropriate use of antimicrobials are available
- Using monitoring and surveillance results to inform action and to update treatment guidelines and essential medicines lists
- Scale-up the number of health care facilities with quality diagnostic tests for infection and AMR
- Mobilising support for this objective to stay on governments' agenda
- Other activities (please specify) ________________________________________________
- None of these
- I don't know

Q33 **Objective 4: For mitigating harm among patients and/or animals infected with AMR organisms:** What activities is your organisation doing towards this?

**In ANIMAL HEALTH:**

- Promoting national legislation to cover all aspects of national manufacture, import, marketing authorization, control of safety, quality and efficacy and distribution of antimicrobial products
- Checking enforcement processes and control are in place to ensure compliance with legislation
- Promoting biosecurity measures and practices to reduce the need for use of antimicrobials and in case of occurrence of an infection, it is contained
- Implementing practices on responsible and prudent use in animals and plants in line with Codex and OIE standards.
- Scale-up responsible practices
- Mobilising support for this objective to stay on governments' agenda
- Other activities (please specify) ________________________________________________
- None of these
- I don't know

Q34 **Objective 4: For mitigating harm among patients and/or animals infected with AMR organisms:** What activities is your organisation doing towards this?

**In the ENVIRONMENT and FOOD PRODUCTION & FOOD SAFETY:**

- Promoting national legislation/ regulations to control discharge of wastewater from health facilities, manure from animals, and industrial effluent to the environment.
- Implementing practices that limit discharge of antimicrobial residues into the environment, including in municipal and pharmaceutical industry waste and wastewater
- Checking enforcement processes and control are in place to ensure compliance with legislation
- Implementing monitoring practices for compliance and enforcement of national legislation/ regulations to prevent contamination of the environment with antimicrobials
- Scale-up responsible practices
- Mobilising support for this objective to stay on governments' agenda
- Other activities (please specify) ________________________________________________
- None of these
- I don't know

| Page Break |  |
| --- | --- |

Q35 **Monitoring activities**
 
The next five questions aim to assess how your organisation monitors the deployment of the activities you responded in the previous section.

Q: Does your organisation have its own strategy for AMR control in Africa?

- Yes
- No

Q36 Is this strategy publicly available?

- Yes
- No

Q37 Does your organisation systematically monitor most of its activities (as responded in the previous section) against its own strategy?

- Yes
- No
- I don't know

Q38 Does your organisation systematically monitor most of its activities (as responded in the previous 'Activities' section) against an overarching strategic framework such as Africa CDC Framework, or National Action Plans?

- Yes, please specify the framework ________________________________________________
- No

Q39 Against the four objectives of Africa CDC AMR Framework (left column), please provide the monitoring metric that you most consistently track to inform the progress of your operations. 


[Please also provide in the right column the frequency of monitoring this metric.  If you do not know this, please put an 'NA' in the box. If progress is not documented, please put an 'X' in this box]

|  | Monitoring metric | Frequency [weekly, monthly, every 6 months, annually, do not monitor) |
| --- | --- | --- |
| Improve surveillance of AMR organisms among humans and animals |  |  |
| Delay emergence of AMR |  |  |
| Limit transmission of AMR |  |  |
| Mitigate harm among patients infected with AMR organisms |  |  |
| Progress is not documented |  |  |

**End of Block: Section 4: ACTIVITIES**

**Start of Block: Section 5: RESOURCES**

Q40 **Section 5: Resources and training needed**
 
These questions aim to gather information on the types of resources and training which are needed by CSOs.
  Which of the following **resources** you would prefer to support your AMR activities?

 [Please choose one of option or write down your own choice]

- Planning tools (such as stakeholder analysis)
- Targeting tools (such as websites, blogs, and media engagement)
- Monitoring and evaluation tools (such as Outcome Mapping)
- Information on national and global AMR policies and plans
- Access to best practice examples/ case studies
- Technical guidance (please specify example) ______________________________________
- Access to data and scientific reports
- Networking opportunities
- Training in basic principles of AMR
- Other [please specify] ________________________________________________

| 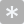 |
| --- |

Q41 What areas of **training** does your organisation need to carry out these activities?

[Please choose **only 3 options** and rank them. With 1 being the highest need, 3 being the least.  You can also add an additional training area]

______ Understand the development and main causes of AMR

______ Understand the basic principles of infection prevention and control (IPC), i.e. hand hygiene to prevent transmission of infections and health-associated infections (HAIs)

______ Understand the impact of resistance on choice of antimicrobial therapy for treating infections

______ Understand the morbidity, mortality and economic threat of AMR to human health

______ Understand the importance of optimising use of antimicrobials in the human and animal sectors to prevent development of resistance

______ Training in responsible antimicrobial prescribing practices

______ Training in communication to empower health-care providers to challenge misuse or overuse of antibiotics

______ Training in communication to influence behaviour change in all relevant sectors

______ Understand antimicrobial use in food-animal production. Problems, solutions, challenges

______ Understand the roles and responsibilities of different stakeholders in antimicrobial stewardship teams. Members could include, but are not limited to, the roles of physicians, pharmacists, infection preventionists, microbiologists, nurses and others

______ Understand local AMR epidemiology, resistance and susceptibility patterns and use of guidelines

______ Understand the diagnostic role of the microbiology laboratory in detecting infections, resistance patterns, guiding patient management and informing AMR control strategies

______ Understand the potential for cost savings and health gains associated with effective infection control and appropriate antimicrobial use

______ Training in digital skills and online-advocacy methods

______ Training in skills such as: communication of public messages, monitoring and evaluation, proposal writing

______ Other [please specify]

**End of Block: Section 5: RESOURCES**

**Start of Block: Section 6: Information sharing**

Q42 **Section 6: Information sharing** 

The two questions in this section aim to inform how information can be shared amongst different stakeholders, as well as to help improve information sharing activities.

Q: Through which of the following platforms (in the right columns) do you currently receive information about AMR?

[Please respond for each of the stakeholder types presented (left column)]

|  | WhatsApp | Facebook/ Twitter | Online forums | Websites | Email | Newsletters | Virtual online meetings | Conferences | Do not receive information from this stakeholder |
| --- | --- | --- | --- | --- | --- | --- | --- | --- | --- |
| Local authorities |  |  |  |  |  |  |  |  |  |
| National authorities |  |  |  |  |  |  |  |  |  |
| Other CSOs |  |  |  |  |  |  |  |  |  |
| Private sector |  |  |  |  |  |  |  |  |  |
| Informal (word of mouth/ individuals) |  |  |  |  |  |  |  |  |  |

Q43 Is there another platform you receive information about AMR? [optional answer]

________________________________________________________________

Q44 How would you like to see information disseminated about the AMR-focused activities which CSOs are doing?

- Annual report
- Bi-annual report
- Presentation at conference
- Website post
- Social media post (e.g. Twitter, Facebook, LinkedIn)
- Other (please specify) ________________________________________________

**End of Block: Section 6: Information sharing**

**Start of Block: Closing**

Q45 Before the survey ends, is there anything you would like to share? [optional answer]

________________________________________________________________

________________________________________________________________

________________________________________________________________

________________________________________________________________

________________________________________________________________

Q46 Please kindly provide your email, in case of any follow-up questions to the survey. This email address will not be shared. Thank you.

________________________________________________________________

**End of Block: Closing**

### SM3: Investment

Table 5. The level of investment, or cost of operations, against AMR in the last financial year (in US$)

| Amount | % | Count |
| --- | --- | --- |
| Less than $50,000 | 40% | 14 |
| Between $51,000 - $100,000 | 3% | 1 |
| Between $101,000 - $200,000 | 6% | 2 |
| Between $201,000 - $500,000 | 3% | 1 |
| More than $500,000 | 0% | 0 |
| Nothing invested on AMR | 31% | 11 |
| I don't know | 17.% | 6 |
| Total | 100% | 35 |

### SM4: Supplementary graphs: Graphs 1-4

Graph 1. Objective 1: Improve surveillance of AMR organisms among humans and animals^[[1]](#footnote-1)^

*** **Most** chosen activities: *campaigning for prescribing practices and appropriate antibiotic use to be monitored in healthcare settings* (69% of the 35 CSOs chose this) and *campaigning for data to be collected and reported on for the total quantity of antimicrobials sold for/used in animals and their intended type of use (therapeutic or growth promotion)* (35% of the 35 CSOs).

**** **Least** chosen activities: *provision of laboratories for surveillance* (14%) and *scaling-up quality clinical diagnostic laboratories with quality assurance programmes* (9%).

****’Other’ activities include: These include advocacy activities; such as, training and awareness campaigns for health professional in the importance of AMR. As well as more technical service provision activities; such as, assessing the capacities of the various sentinel sites to collect samples and analyse them for resistance/sensitivity to antibiotics.*


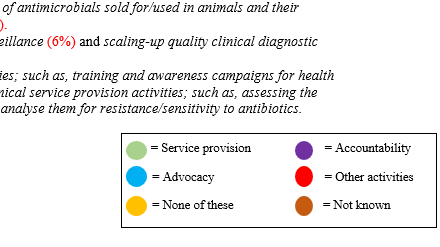
****** *Animal health and food production activities are presented together because of the overlap in the activities of these sectors*

Graph 2. Objective 2. Delay emergence of AMR^[[2]](#footnote-2)^

* **Most** chosen activities overall: *campaigns against the sales of substandard and counterfeit antibiotics (46% of the 35 CSO chose this) and mobilising support for this objective to stay on governments' agenda (43% of CSOs chose this)*

**** **Least** chosen activities overall: *Using surveillance results to inform and scale-up government action and to update treatment guidelines and essential medicines lists (14%)* and *checking enforcement processes and control are in place to ensure compliance with legislation (14%).*

*** ‘***Other****’ activities include: holding trainings on IPC, data management and analysis, and appropriate antimicrobial use. Promoting the use of good quality of antibiotics by setting up quality assurance systems in procurement distribution, storage, and use of antibiotics*

***** Where sectors were not represented in the activities, they are not included in the graph.*


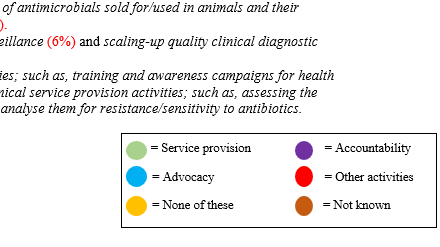


Graph 3. Objective 3. Limit transmission of AMR^[[3]](#footnote-3)^

*** **Most** chosen activity overall was *mobilising the objective to stay on the government’s agenda* **(**46% of the 35 CSO chose this for all three sector-related activities).

**** **Least** chosen activity overall was *ensuring plans and guidance are updated in response to monitoring results* (14% of CSOs)

*** **‘Other’** activities include*: promoting multisectoral collaboration and participation between human health environmental* health *and animal health, especially to strategize against occurrence of diseases, zoonoses and AMR; masterclasses for data management and analysis for AMR to promote IPC; promoting the combination of advocacy and service provision WASH activities.*


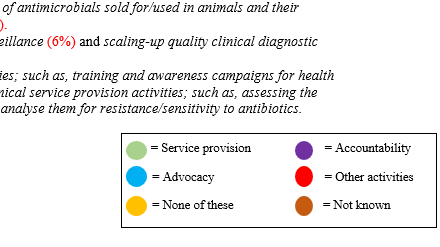


Graph 4. Objective 4. Mitigate harm among patients infected with AMR organisms^[[4]](#footnote-4)^

* **Most** chosen activity *was* *mobilising the objective to stay on the government’s agenda (40% and 37% of CSOs chose this in human and animal & environment related activities, respectively),* and *encouraging governments to enforce a national policy for antimicrobial governance and regulation for appropriate use and campaigning to maintain access to essential antibiotics (51% of CSOs).*

**** **Least** focus was on service-provision activities**:** *using monitoring and surveillance results to inform action and to update treatment guidelines and essential medicines lists (11% of CSOs),* and *scaling-up the number of health care facilities with quality diagnostic tests for infection and AMR* *(17% of CSOs).*

*** **‘Other’** activities: *lobbying for faith-based communities to be involved.*

***** Where sectors were not represented in the activities, they are not included in the graph (e.g. Food safety).*


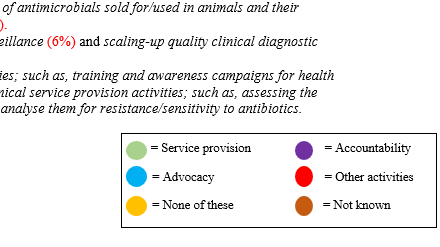


### SM5: Monitoring

Table 6. Example frameworks: Monitoring metrics

| **Yes, please specify the framework** | **Sector of respondent** |
| --- | --- |
| Against SDG 6 on WASH and SDG3 on health, National frameworks on WASH in healthcare facilities and global commitments on WASH in healthcare facilities | Environment |
| We evaluate our performance against our global policy, work plan and the Kenyan action plan for reduction of AMR resistance | Animal |
| National Action Plan against Malaria | Human |

### SM6: Sharing information

Table 7. Preferences for disseminating information about AMR activities amongst CSOs

| **Information sharing methods: Overall group** | **%** | **Count** | **Rank** |
| --- | --- | --- | --- |
| Social media post (e.g. Twitter, Facebook, LinkedIn) | 24.56% | 28 | 1 |
| Presentation at conference | 22.81% | 26 | 2 |
| Website post | 20.18% | 23 | 3 |
| Annual report | 13.16% | 15 | 4 |
| Bi-annual report | 12.28% | 14 | 5 |
| Other (please specify) | 7.02% | 8 | 6 |
| Total | 100% | 114 |  |

1. The bubble sizes directly correlate to the number of times the activity (grouped by coding of activity categories) was selected by the CSOs. As the survey questions were designed using the structure of the Tripartite survey and in relation to Africa CDC Framework objectives, not every objective has activities related to each sector. Comparing sizes of bubbles across the sectors is taken with caution as the sectors have different representation sizes (human (20), animal (10), environment (5). Instead, the sizes of bubbles within the same sector of the organisation can be compared. [↑](#footnote-ref-1)
2. The bubble sizes directly correlate to the number of times the activity (grouped by coding of activity categories) was selected by the CSOs. As the survey questions were designed using the structure of the Tripartite survey and in relation to Africa CDC Framework objectives, not every objective has activities related to each sector. Comparing sizes of bubbles across the sectors is taken with caution as the sectors have different representation sizes (human (20), animal (10), environment (5). Instead, the sizes of bubbles within the same sector of the organisation can be compared. [↑](#footnote-ref-2)
3. The bubble sizes directly correlate to the number of times the activity (grouped by coding of activity categories) was selected by the CSOs. As the survey questions were designed using the structure of the Tripartite survey and in relation to Africa CDC Framework objectives, not every objective has activities related to each sector. Comparing sizes of bubbles across the sectors is taken with caution as the sectors have different representation sizes (human (20), animal (10), environment (5). Instead, the sizes of bubbles within the same sector of the organisation can be compared. [↑](#footnote-ref-3)
4. The bubble sizes directly correlate to the number of times the activity (grouped by coding of activity categories) was selected by the CSOs. As the survey questions were designed using the structure of the Tripartite survey and in relation to Africa CDC Framework objectives, not every objective has activities related to each sector. Comparing sizes of bubbles across the sectors is taken with caution as the sectors have different representation sizes (human (20), animal (10), environment (5). Instead, the sizes of bubbles within the same sector of the organisation can be compared. [↑](#footnote-ref-4)
